# Effectiveness of a Glyceryl Trinitrate (GTN) Patch in Preventing Amputation, Improving Pain Control and Reducing the Size of Tissue Loss for a Patient With Critical Limb-Threatening Ischaemia (CLTI)

**DOI:** 10.7759/cureus.62388

**Published:** 2024-06-14

**Authors:** Muhammad Asghar Butt, Mohammad Mostafizur Rahman Miah, Dani Avabde, Murali Subramaniam

**Affiliations:** 1 Vascular Surgery, United Lincolnshire Hospital NHS Trust, Lincolnshire, GBR; 2 Vascular Surgery, Nottingham University Hospital NHS Trust, Nottingham, GBR

**Keywords:** amputation, diabetic foot, critical limb threatening ischaemia (clti), tissue loss, glyceryl trinitrate (gtn) patch

## Abstract

Background

Foot ulcer is a common complication of poorly controlled diabetes and peripheral vascular disease (PVD). The current standard of treatment for diabetic foot ulcers includes the management of underlying risk factors, wound debridement, use of antibiotics for infection, off-loading with cast, and revascularisation surgery. The glyceryl trinitrate (GTN) patch is currently off-licence in treating PVD or diabetic foot ulcers. This study aims to evaluate the effectiveness of the GTN patch in preventing amputation, improving pain control, and reducing the size of tissue loss (ulcer/gangrene) or localised ischaemic area.

Method

This is a pilot study of 30 patients who were started on the GTN patch from February 2020 to October 2021. Inclusion criteria were patients who have critical limb-threatening ischaemia (CLTI) and with no viable options or are at high risk for revascularisation, both endovascular and open surgery. Patients who were on a GTN patch for less than six weeks at the time of data collection or had unclear outcomes were excluded.

The outcomes were retrospectively collected on prevention of amputation, improvement in pain control, and reduction in tissue loss (the size of ulcer/gangrene) or localised ischaemic area with the use of a GTN patch. The binomial test was used to compare the observed outcome of the GTN patch and the expected outcome, which was assumed to be 50% in this study.

Results

Ninety-three per cent (93%) of the patients who had GTN patches successfully avoided amputation (p<0.0001). Eighty-four per cent (84%) of patients reported better pain control (p=0.0022) and improvement in the size of ulcer/gangrene/localised ischaemic areas (p=0.0005).

Conclusion

The GTN patch is effective in preventing amputation, improving pain control, and reducing the size of ulcer/gangrene/localised ischaemic areas in patients who have end-stage CLTI and no viable options or who are at high risk for revascularisation surgery.

## Introduction

Diabetic foot disease is a common complication faced by diabetic patients. According to a systematic review and meta-analysis conducted in 2016, the worldwide prevalence of diabetic foot ulcers was 6.3% [1]. In England, this figure stood at 2-2.5% of the diabetic population and this translated to approximately 60,000 to 75,000 people [2]. People with diabetes are 23 times more likely to have either toe, foot or leg amputation than those without diabetes [2]. The quality of life of diabetic patients with an amputation is severely impacted due to impaired mobility and the risk of recurrent ulcers. In 2014-2015, the care of diabetic foot ulcers and amputation cost NHS England about £972 million to £1.13 billion [2]. Peripheral vascular disease often coexists with uncontrolled diabetes and contributes to the development and poor healing of foot ulcers.

In the current practice, the standard of treatment of diabetic foot ulcers includes surgical wound debridement, use of antibiotics for bone or wound infection, off-loading of the ulcer with cast and revascularisation surgery when indicated [3]. The management of underlying risk factors, such as poorly controlled diabetes, hypertension, and peripheral vascular disease, is equally important. Antiplatelets or anticoagulants, lifestyle modifications, such as dietary control, smoking cessation, being physically active, and tight control of the blood glucose, blood pressure, and cholesterol level will help improve the underlying peripheral vascular disease [4].

Some newer therapies that have been suggested for the management of diabetic foot ulcers include hyperbaric oxygen therapy (HBOT), the use of advanced wound care products, which contain matrix metalloproteinase regulators or hyaluronic acid, stem cell therapy and negative-pressure wound therapy (NPWT) [3]. HBOT enhances collagen synthesis and maturation, fibroblast proliferation, promotes angiogenesis and increases the leucocyte bacterial-killing capacity [3,5]. Stem cells have tremendous potential to differentiate and evolve into differentiated cell types. Clinical benefits reported from the use of stem cell therapy include improvement in ankle-brachial pressure index (ABPI), transcutaneous partial pressure of oxygen (TcPO2) and reduction in pain and amputation rates [6]. NPWT, on the other hand, will optimise the blood circulation to the wound and remove the inflammatory exudates and bacteria from the wound area [3].

The newer therapies mentioned above are mainly used as adjunct therapy to the current conservative therapy when optimal ulcer healing is not achieved. The desirable outcome of adding these adjunct therapies is to avoid limb amputation. One of the newer therapies that will be discussed in detail in this study is glyceryl trinitrate (GTN).

GTN, also known as nitroglycerin, was first produced by an Italian chemist, Ascanio Sobrero, and was linked to causing severe headaches. The work of Ascanio Sobrero was later advanced by Alfred Nobel with the invention of dynamites [7]. On the other hand, the development of medical use of GTN was mainly built on the discovery of the vasodilatory effect of nitric oxide with mediated increased cyclic guanosine monophosphate (cGMP) and nitric oxide is the endothelium-derived relaxing factor [8]. A Nobel Prize in physiology or medicine was awarded to Robert Furchgott, Ferid Murad and Loius Ignarro in 1998 based on this discovery [7].

A GTN transdermal patch has been widely used to prevent angina and maintain venous patency as per the British National Formulary (BNF) [9]. However, it is not licenced to treat peripheral vascular disease or diabetic foot ulcers. When applied to the skin, glyceryl trinitrate is slowly absorbed through the skin into the bloodstream. It is denitrated to form free radical nitrite oxide in the blood vessel smooth muscle cells, which subsequently helps dilate the blood vessel and improves the blood circulation to the area [4,10]. As such, the GTN patch should be able to improve blood circulation in peripheral vascular disease, promote ulcer healing and reduce the likelihood of amputation.

Having said that, there were not many studies available to date to investigate the effectiveness of GTN patches in improving peripheral vascular disease or diabetic foot ulcers. A multicenter, prospective randomised controlled trial done in 2018 revealed that the use of nitric oxide wound dressing was able to reduce the size of ulcers twice faster at week 12 compared to those without the dressing [11]. An improvement in the healing of foot ulcers with the use of the GTN patch was also obtained by another research carried out by Sylvia McAra in 2015 [12].

Due to the limited evidence available, there is a need to do more studies on the effectiveness of the GTN patch. The primary aim of this study is to investigate the effectiveness of the GTN patch in avoiding amputation in patients who have severe peripheral vascular disease and are high-risk surgical candidates. The secondary objective of this study is to determine the effectiveness of the GTN patch in improving pain control and reducing the size of ulcers, gangrene, and localised ischaemic areas on the foot.

## Materials and methods

One of the senior authors of our study has started using GTN patches in patients with end-stage chronic limb-threatening ischaemia (CLTI) and no viable options or very high risk of revascularisation, both endovascular and open surgery, since 2008. Currently, the use of GTN patches in these patients is off-licence in the British National Formulary (BNF). The approval from the pharmacist in ULHT was obtained before the introduction of GTN patch intervention in this group of patients. The study was carried out in accordance with the ethical standards set out by the local committee and the Declaration of Helsinki.

Thirty patients who were started on a GTN patch from February 2020 until October 2021 for peripheral vascular disease in a district general hospital in the United Kingdom were identified. Inclusion criteria for this study were patients who have end-stage chronic limb-threatening ischaemia (CLTI) and with no viable options or are at high risk for revascularisation procedures such as endovascular and open surgery. Patients who were on a GTN patch for less than six weeks at the time of data collection or had unclear outcomes were excluded. Patients’ clinic letters and discharge letters were used to collect the follow-up data retrospectively up until mid-October 2021 mainly on their progress after being started on the GTN patch. The duration of GTN patch use ranged from 6 weeks to 20 months among our patients.

The data on the progression of the size of ulcer/gangrene /localised ischaemic area, requirement of amputation, as well as the severity of pain secondary to ulcer/claudication/rest pain, were collected retrospectively from the hospital database. Patients were determined as successfully avoiding amputation if they did not proceed to have an amputation or die of complications related to their peripheral vascular disease after starting on the GTN patch during the data collection period, which was until the second week of October 2021.

Among the 30 patients identified in this study, patients who reported pain (n=19) or had ulcer/gangrene/localised ischaemic areas (n=25) were further selected to evaluate the secondary outcome of the GTN patch. Patients who reported improvement in the distance of claudication/rest pain/pain from an ulcer were classified as responders to the GTN patch. The examination findings of the patients who were treated for ulcers, gangrene, or localised ischaemic areas documented in follow-up clinic letters were used to evaluate the effectiveness of the GTN patch on secondary outcomes.

Statistical analysis was done to compare the distribution of patients successfully prevented from amputation with the GTN patch intervention to the expected distribution of patients avoiding amputation with conventional conservative management. The theoretically expected proportion of patients avoiding amputation with the use of a GTN patch was assumed to be 50%. Statistical analysis was carried out using Microsoft Excel (Microsoft Corp., Redmond, WA, USA) and GraphPad Prism software (www.graphpad.com). The binomial test was used to compare the observed and expected distributions. A p-value <0.05 is considered statistically significant.

## Results

Thirty patients were included in this study. These patients had severe peripheral vascular disease or diabetic foot ulcer and had received a GTN patch for at least six weeks. The male patients accounted for the majority (80%, n=24) of the sample size compared to their female counterparts. The age of patients ranged from 61 to 94 years, the mean age was 78 years, and the median age was 79. Many of the patients had hypertension (83%) and/or diabetes (67%). However, only a small proportion (17%) of the patients have a background of ischaemic heart disease (Table 1).

**Table 1:** Patient’s demographic and comorbidities

	Criteria	Number of patients	proportion
Gender	Male	24	80%
	Female	6	20%
Age	60-69 year	5	17%
	70-79 year	12	40%
	80-89 year	12	40%
	90-99 year	1	3%
Co-morbidities	Hypertension	25	83%
	Diabetes	20	67%
	Ischaemic heart disease	5	17%

A GTN patch was deemed effective in preventing amputation, pain control, and tissue loss (ulcer/gangrene) or the size of the localised ischaemic area in patients with CLTI (Table 2).

**Table 2:** Outcome of the GTN patch on preventing amputation, pain control, and tissue loss (ulcer/gangrene) or localised ischaemic area size * 19 out of 30 patients reported pain secondary to claudication, rest pain or localised ischaemic area before starting on GTN patch treatment ** 25 out of 30 patients had tissue loss (ulcer/gangrene) or localised ischaemic area before starting on GTN patch treatment GTN: glyceryl trinitrate

Outcome	Total (n)	Yes	No
Prevention of amputation	30	28	2
Improvement of pain control	19*	16	3
Improvement of ulcer/gangrene/localised ischaemic area size	25**	21	4

The vast majority (93%, n=28) of the patients who had the GTN patch intervention avoided the need for amputation (p=<0.0001) (Table 3).

**Table 3:** Effect of a GTN patch intervention on preventing amputation GTN: glyceryl trinitrate

Prevention of amputation	Expected frequency (%)	Observed frequency (%)	95% Confidence interval of observed %	p-value
Yes	15 (50%）	28 (93.33%)	78.68 - 98.82	<0.0001
No	15 (50%)	2 (6.67%）	1.18 - 21.32	

Most of the patients (84%, n=16) of the patients who experienced prior pain related to peripheral vascular disease showed significant improvement in pain control (p=0.0022) with the GTN patch (Table 4).

**Table 4:** Effect of glyceryl trinitrate (GTN) patch intervention on improving pain control

Improvement of pain control	Expected Frequency (%)	Observed frequency (%)	95% Confidence interval of observed %	p-value
Yes	9.5 (50%)	16 (84.21%)	62.43 - 94.48	0.0022
No	9.5 (50%)	3 (15.79%)	5.52 - 37.57	

A large proportion of patients (84%, n=21) who had tissue loss (ulcers/gangrene) or localised ischaemic area on foot prior to starting the GTN patch, were found to have a significant reduction in size/depth (p=0.0005) or achieved complete healing (Table 5).

**Table 5:** Effect of GTN patch intervention on reducing ulcer/gangrene/localised ischaemic area GTN: glyceryl trinitrate

Improvement of ulcer/gangrene/localised ischaemic area	Expected frequency (%)	Observed frequency (%)	95% confidence interval of observed %	p-value
Yes	12.5 (50%)	21 (84%)	66.35 - 93.6	0.0005
No	12.5 (50%)	4 (16%)	6.40 - 34.65	

## Discussion

This is a pilot study to evaluate the effectiveness of a GTN patch as an adjunct therapy to avoid amputation in patients with significant peripheral vascular disease and who have a very high risk of undergoing major revascularisation surgery. Besides the vasodilatation properties of GTN, there are other effects of nitric oxide, such as anti-inflammatory effect, antimicrobial effect, inhibition of platelet aggregations and modulation of mitochondrial functions [13,14]. These effects can be extrapolated in the use of wound and ulcer healing. Several studies have been done to evaluate the use of topical GTN or nitric oxide donors to treat digital ulcers secondary to systemic sclerosis and chronic ulcers due to leishmaniasis [15,16].

The use of GTN patches is an additional intervention to conventional conservative management, which includes antiplatelet and statin, localised debridement, prolonged antibiotics course for infected ulcers and regular dressings.

This study showed that the GTN patch was effective in preventing amputation, pain control, and tissue loss (ulcer/gangrene) or the size of the localised ischaemic area and thus avoided the need for amputation. Most of the patients showed improvement in pain control with a GTN patch. A large proportion of patients who had tissue loss were found to have a reduction in size/depth or achieved complete healing after using a GTN patch.

Based on the previous meta-analysis diabetic neuropathic ulcers had a mean healing rate of 42% at 20 weeks with standard care therapy alone [17]. The outcome has improved when compared to a study in 1999 (mean healing rate = 31%) [16]. We have hence set the assumed expected proportions of patients with good outcomes with standard therapy as 50%. When compared to the expected distribution, a significant improvement in the prevention of amputation with the introduction of GTN patch was shown in our study (p<0.0001). Similarly, GTN patch intervention also had a significant positive impact on pain control (p= 0.0022) and healing of ulcer/gangrene/localised ischaemic area (p=0.0005). The result of improvement of tissue loss (ulcer/gangrene) or localised ischaemic area size was consistent with the results from the previous study [18].

Out of the 30 patients who had the GTN patch, one patient discontinued the use of the GTN patch after six to eight weeks due to blisters and increased leg swelling. There may be limited uptake of reported side effects due to not being actively sought for during clinic visits or data collection.

The sample size in our study was limited by the off-licence use of GTN patches in peripheral vascular disease or treatment of diabetic foot ulcers. In addition, there was also no control group in this study. Further prospective studies with a larger sample size and the addition of a control group could be conducted to further evaluate the effectiveness of the GTN patch.

## Conclusions

A foot ulcer is a common complication of poorly controlled diabetes and peripheral vascular disease. It is a high cost for the National Health Service (NHS) to manage each year. The current gold standard treatment for diabetic foot ulcers includes the management of underlying risk factors such as diabetes, hypertension or peripheral vascular disease, surgical wound debridement, use of antibiotics for infection, off-loading of the ulcer with cast, and revascularisation surgery when indicated. The GTN patch is currently not licenced under BNF to manage peripheral vascular disease or diabetic foot ulcers.

Based on this study, the use of a GTN patch has shown a promising result in preventing amputation, improving pain control, and reducing tissue loss (ulcer/gangrene) or localised ischaemic area size in patients who have severe peripheral vascular disease and who are at high risk for major revascularisation surgery. A GTN patch could be considered an additional intervention in this patient group, on top of the current conservative management. Further studies with larger sample sizes and repeat studies to directly compare patients’ outcomes with or without the intervention of the GTN patch would be useful to further establish the effectiveness of the GTN patch.
